# Effectiveness of the Hospital Learning Center (Queen Sirikit National Institute of Child Health): Satisfaction with service and parents’ attitudes towards children’s illness

**DOI:** 10.12688/f1000research.18846.1

**Published:** 2019-09-10

**Authors:** Adidsuda Fuengfoo, Sija Leelathanaporn, Thanyaporn Mekrungcharas, Kim Sakulnoom, Sumitra Owjinda, Piyanat Noipong, Suphawan Srinuan, Sarunya Kumjaroen, Natchanan Phonok

**Affiliations:** 1 Pediatric Department, Queen Sirikit National Institute of Child Health, Bangkok, 10400, Thailand

**Keywords:** Children with illness, Education service, School Engagement

## Abstract

**Background: **All children, whether healthy or ill, should have access to equal educational opportunities. Healthcare institutions and hospitals have been approved to work with schools to establish learning centers to provide education to sick children. This study has been conducted to develop a practical model for learning centers in hospitals across Thailand to create equality and ensure valuable human resources for the future. The main goal of this study was to evaluate the effectiveness of a hospital learning center for continuing education of child patients and to determine the factors that are most appropriate study plans, the parents’ attitude about their child’s illness, and the children’s satisfaction with the learning center.

**Methods: **The total sample population was 400, consisting of 200 parents and 200 child patients aged 4 to 18 years. The respondents were given a questionnaire to obtain their feedback using a Likert scale.

**Results: **The most common child patients were those with chronic illness followed by those with common illnesses, and lastly children with developmental problems. All 200 children received continuing education; 20 child patients (10%) who had been evaluated received a modified education plan. After analyzing the results of satisfaction with the learning center, the scores ranged from 4.21 to 5.00 (mean = 4.28, SD = 0.62).

**Conclusions:** Sick children can continue their education at the hospital learning center in Queen Sirikit National Institute of Child Health. Study plans can be modified to suit children with chronic illnesses and developmental problems, children in primary and secondary school, and those requiring prolonged hospitalization. Parents in the study had appropriate attitudes about the disease and education of their children. Sick children gave the highest ratings showing extremely high satisfaction with the hospital learning center.

## Introduction

Information technology, social networking, and the creation of a global economy continue to develop rapidly, but education is still the primary basis of child development and growth. All children, whether sick or healthy should receive equal education. The number of children living with chronic conditions has steadily risen as improvements ^1^ in medication and pediatric care for chronic illness have resulted in more children with previously lethal conditions now surviving into adulthood and beyond ^1, 2^. The prevalence of children with chronic disease varies depending on the definition used. One survey found that the prevalence of chronic health condition in children was 13-27 percent ^3^. The number of children in Thailand with chronic disease is not clearly defined because chronic diseases can develop slowly over time ^4, 5^; some are incurable, requiring long-term treatment with adverse effects on the patient’s and family’s daily activities ^5, 6^. Generally, children with chronic conditions have lengthy hospital stays and require continued follow-up after going home, causing major changes in the lives of patients and families. Some patients do well in home care but their condition may be too serious to permit them to return to school. Some lose a lot of school days, which prevents them from sharing social experiences ^7, 8^. The Convention on the Rights of the Child recognized education as a legal right of every child on the basis of equal opportunity ^9^. As a member of the United Nations, Thailand has adopted a law that states “education is not limited the classroom”. Hospital and health care institutions have been approved to establish learning centers in the hospital to provide education for ill children under collaboration between hospitals and schools. Queen Sirikit National Institute of Child Health (QSNICH) is working under a project called “The Information Technology Project under the Initiative of Her Royal Highness Princess Maha Chakri Sirinhorn”, by using technology to support learning with the help of multidisciplinary team, which increases the opportunity to connect with education and society.

The Learning Center in the Hospital at QSNICH has been operating for 20 years. A previous study ^10^ identified educational and social perspectives of child patients that influenced psychological conditions and adaptation to illness but did not address the effectiveness of the learning center. With collaboration between various sectors at the ministry level, this study has been conducted to determine the effectiveness of the center and to encourage continuing and appropriate education plans for child patients, as well as to identify the factors that relate to modification of education plans. The results of this study will be useful for developing education models of hospital learning centers for implementation at hospitals across Thailand to provide educational equality and valuable human resources for the future.

## Objectives

The primary objective of this study was to evaluate the effectiveness of the hospital learning center and to determine appropriate plans for continuing and improving the education of child patients.

The secondary objective was to identify the factors involved in the adjustment of educational plans, assess the parents’ attitudes about their child’s illness, and rate the satisfaction of the children with the learning center.

## Methods

### Study design

A cross-sectional survey was conducted to obtain information about the parents and the children who entered the learning center at QSNICH, Bangkok, Thailand between January 1 and December 31, 2018.

### Sample size and selection

The total population was 400 persons, consisting of 200 parents and 200 sick children from the learning center in hospital at the QSNICH from January 1, 2018 to December 31, 2018. The sample size was calculated based on a previously described method ^11^ by reviewing the number of children who returned to school (80% of all children), given p = 0.8, N = 1000. The computed result gave a sample size of 198 which led to the number of 200 children and 200 parents, giving 400 persons in total. The inclusion criteria were sick children at the learning center aged between 4 and 18 years, sick children who visited the learning center more than twice and sick children with critical illnesses at the hospital or home who still having continual treatment. The exclusion criterion was sick children at terminal stage of life or having critical illnesses. Informed consent for study participation was obtained from child patients and parents.

### Data collection

Researchers used systematic random sampling in which the children on the name list at the learning center, QSNICH were selected in order. If any respondents refused to answer the questions, the latter name would be selected. After giving consent, the researcher started interviewing parents and sick children. For children from 4 to 7 years of age, parents were asked to join the interview ^12^. The answers were noted, checked, and recorded for data analysis.

The questionnaire was divided into four parts; demographic data of parents, relationship between parents and children, children’s personal information and satisfaction with service at learning center using a Likert scale. The rating scores were divided into five levels. The questionnaire used, alongside an English translation, is available as *Extended data* ^13^.

The variables associated in this research included parents and children’s relationship, educational level, parent’s occupation, family income, home town, severity of diseases, period of hospitalization, and satisfaction with service at the learning center.

### Statistical analysis

Levels of significance based on satisfaction scores of the learning center at the hospital and were divided into five levels: Strongly agree (4.21–5.00 points), agree (3.41–4.20 points), uncertain (2.61–3.40), disagree (1.81–2.60), strongly disagree (1.00–1.80). P < 0.05 was considered to indicate statistical significance.

DataFax was used for data management. Descriptive statistics, including percent, mean and standard deviation were used for analyzing population data and SAS 9.4 software was used to calculate frequency, percentage, mean and standard deviation to measure attitude and satisfaction of parents and children at the center. Data on satisfaction was divided into 5 levels: strongly agree, uncertain, disagree, strongly disagree based on statistical test results. Chi-square test and t-test were used to assess variables associated with continuation of education at the learning center.

### Ethical statement

This study was been approved by the Office for Ethics in Human Research, Queen Sirikit National Institute of Child Health in 2018 (ref. REC.010/2562), and was conducted in accordance with The Declaration of Helsinki

## Results

Responses to each question of the survey from each participant is available as *Underlying data* ^14^.

### Background of caregivers

The study categorized the children into three groups: those with common illness, those with chronic conditions and disabled children. The other population studied was the 200 caregivers, the largest percentage of which was the biological parents of the children at 87.9%, 91.5% and 93.3% for the three groups, respectively; caregivers who are relatives were caregivers for 12.1%, 8.5% and 6.7%, respectively. Neither foster parents nor teachers were guardians. The majority of caregivers were female (90%, 90% and 53% for those with common illness, those with chronic conditions and disabled children, respectively), of Thai nationality (100%, 100%, and 93%, respectively) and Buddhist religion (97%, 100%, and 93%, respectively); the non-Thai caregivers were from Myanmar and other religions included Islam and Christianity. Most parents were 31 to 40 years of age (46.1%, 44.7%, and 53.3% for those with common illness, those with chronic conditions and disabled children, respectively). Those 41 to 50 years old constituted 23%, 39%, and 40%, respectively, while parents who were 21 to 30 years old were involved with only 21%, 9%, and 0% of the children, respectively. Most parents had a bachelor’s degree or vocational education, but a small percentage went to secondary school. Occupations of most parents were in sales and agriculture or laborers. Most were married, separated, or divorced. The majority had income in the range of THB 10,000-30,000, with the rest earning THB 5,000-10,000, and only a small proportion receiving more than THB 30,000 or less than THB 5,000. The study families in general were home owners, while some rented a house or apartment and lived in the central part of Thailand. Only a few lived with relatives.

According to Table 1, for the 200 child patients, 91 caregivers (45.5%) were parents of children with common illnesses, 94 (47%) were parents of children with chronic illnesses and 15 (7.5%) were parents of children with disabilities. In general, parents of the three categories of sick children rated each question similarly. Regarding the severity of the illnesses, 12.0% rated them at the minimum level, 46.5% at the moderate level, and 39.5% at the high level. Whenever the patients get ill, 74.5% of the caregivers always reported it to school authorities while 20.5% frequently did so. About 34.0% were very confident that their children got along well with their peers, 31.0% were moderately confident and 29% highly confident. Similarly, 44.5% of caregivers attempted to help their children with school work at a moderate level, while 23.5% had tried at a higher level. Overall, 34.5% often explained the classwork to their children and 33.5% did it all the time. About 11% were moderately confident that the school arranged good study plans for their children, 47% were moderately confident and 38.5% were highly confident. Overall, 44% of the child patients talked about what happened in their school and 18.5% did it all the time. Regarding the caregivers’ confidence to help their children to control their emotions, about 42% were very confident, and about 17% were moderately confident and 34% were extremely confident. In total, 91.37% of caregivers took their children to school on a daily basis.

**Table 1.** Relationship between parents and children.

Parent’s Feedback	Common Illness, n (%)	Chronic Illness, n (%)	Disabled Children, n (%)	Total, n (%)
Total	91(45.5%)	94 (47%)	15 (7.5%)	200 (100%)
Severity of illness while receiving education at the center				
None	3 (3.30)	1 (1.06)	0 (0.00)	4 (2.00)
Minor	18 (19.78)	3 (3.19)	3 (20.00)	24 (12.00)
Moderate	45 (49.45)	36 (38.30)	12 (80.00)	93 (46.50)
Major	21 (23.08)	36 (38.30)	0 (0.00)	57 (28.50)
Extreme	4 (4.40)	18 (19.15)	0 (0.00)	22 (11.00)
How often do you inform the teacher when your child is sick?				
Never	1 (1.10)	0 (0.00)	0 (0.00)	1 (0.50)
Almost never	5 (5.49)	4 (4.26)	0 (0.00)	9 (4.50)
Sometimes	10 (10.99)	10 (10.64)	2 (13.33)	22 (11.00)
Often	11 (12.09)	3 (3.19)	5 (33.33)	19 (9.50)
Always	64 (70.33)	77 (81.91)	8 (53.33)	149 (74.50)
How much confidence do you have towards the ability of your child building relationships with friends				
Not at all confident	0 (0.00)	0 (0.00)	0 (0.00)	0 (0.00)
Low confidence	7 (7.69)	3 (3.19)	2 (13.33)	12 (6.00)
Moderately confident	20 (21.98)	35 (37.23)	7 (46.67)	62 (31.00)
Very confident	34 (37.36)	29 (30.85)	5 (33.33)	68 (34.00)
Extremely confident	30 (32.97)	27 (28.72)	1 (6.67)	58 (29.00)
How much effort do you provide your child for self-learning?				
No effort	1 (1.10)	1 (1.06)	0 (0.00)	2 (1.00)
Little effort	15 (16.48)	17 (18.09)	2 (13.33)	34 (17.00)
Much effort	12 (13.19)	14 (14.89)	2 (13.33)	28 (14.00)
Great effort	40 (43.96)	40 (42.55)	9 (60.00)	89 (44.50)
Extreme effort	23 (25.27)	22 (23.40)	2 (13.33)	47 (23.50)
How often do you explain the lesson to your child?				
Almost never	0 (0.00)	4 (4.26)	0 (0.00)	4 (2.00)
Sometimes	7 (7.69)	7 (7.45)	1 (6.67)	15 (7.50)
Often	22 (24.18)	21 (22.34)	2 (13.33)	45 (22.50)
Always	31 (34.07)	30 (31.91)	8 (53.33)	69 (34.50)
Almost never	31 (34.07)	32 (34.04)	4 (26.67)	67 (33.50)
Are you confident that the school provides appropriate education to your child?				
Not at all confident	0 (0.00)	0 (0.00)	0 (0.00)	0 (0.00)
Low confidence	6 (6.59)	1 (1.06)	0 (0.00)	7 (3.50)
Moderate confidence	8 (8.79)	11 (11.70)	3 (20.00)	22 (11.00)
Very confident	38 (41.76)	48 (51.06)	8 (53.33)	94 (47.00)
Extremely confident	39 (42.86)	34 (36.17)	4 (26.67)	77 (38.50)
Do your children tell you about life at school?				
Never	0(0.00)	2(2.13)	0(0.00)	2(1.00)
Almost never	9(9.89)	7(7.45)	3(20.00)	19(9.50)
Sometimes	27(29.67)	26(27.66)	1(6.67)	54(27.00)
Frequently	35(38.46)	42(44.68)	11(73.33)	88(44.00)
Always	20(21.98)	17(18.09)	0(0.00)	37(18.50)
Are you confident about helping your child manage emotion appropriately?				
Not at all confident	0 (0.00)	0 (0.00)	0 (0.00)	0 (0.00)
Low confidence	8 (8.79)	4 (4.26)	2 (13.33)	14 (7.00)
Moderate confidence	15 (16.48)	15 (15.96)	4 (26.67)	34 (17.00)
Very confident	37 (40.66)	41 (43.62)	6 (40.00)	84 (42.00)
Extremely confident	31 (34.07)	34 (36.17)	3 (20.00)	68 (34.00)
Do you usually pick up and drop off your child at school?				
Yes	65 (71.43)	61 (64.89)	13 (86.67)	139 (69.50)
No	26 (28.57)	33 (35.11)	2 (13.33)	61 (30.50)
If yes, how do you take your child to school?				
Pick up and drop off	62 (95.38)	55 (90.16)	10 (76.92)	127 (91.37)
Drop off, wait until done, take children home	0 (0.00)	0 (0.00)	0 (0.00)	0 (0.00)
Either pick up or drop off	3 (4.62)	6 (9.84)	3 (23.08)	12 (8.63)

### Children with common illnesses

As shown in Table 2, 23.1% of children with common illness were in kindergarten, 64.8% in primary school and 10.9% in secondary school before entering the learning center. After discharge, 20.8% were in kindergarten, 67.0% were in primary school and 10.9% were in secondary school. The distances from home to school before and after attending the learning center did not differ. Approximately, 16.5% lived less than 1 km away, 60.4% lived 1–5 km away and 23.1% lived the same distance as before entering the learning center. The data also showed that 29.7% went to school by bicycle or motorcycle, 39.6% by personal car, and 28.6% by public transportation; the mean of transportation was not much different from before and after entering learning center.

**Table 2.** Patient’s information before and after entering the learning center.

Educational Level (Children)	Before entering the learning center in hospital	After entering the learning center in hospital
	N (%)	N (%)
Children with Common Illness	N= 91	N= 91
Level of Education		
Kindergarten	21 (23.08)	19 (20.88)
Primary School	59 (64.84)	61 (67.03)
High School	10 (10.99)	10 (10.99)
Vocational Education	1 (1.10)	1 (1.10)
Non-formal Education	0 (0.00)	0 (0.00)
Others	0 (0.00)	0 (0.00)
Distance from Home to School		
< 1 km.	15 (16.48)	15 (16.48)
1–5 km.	55 (60.44)	55 (60.44)
> 5 km.	21 (23.08)	21 (23.08)
Transportation to School		
Walk	1 (1.10)	1 (1.10)
Bicycle/Motorcycle	27 (29.67)	27 (29.67)
Family vehicle	36 (39.56)	36 (39.56)
Others	26 (28.57)	26 (28.57)
Boarding school	1 (1.10)	1 (1.10)
Children with Chronic Illness	N= 94	N= 94
Level of Education		
Kindergarten	10 (10.64)	10 (10.64)
Primary School	64 (68.09)	59 (62.77)
High School	20 (21.28)	20 (21.28)
Vocational Education	0 (0.00)	0 (0.00)
Non-formal Education	0 (0.00)	4 (4.26)
Others	0 (0.00)	0 (0.00)
Distance from home to school		
< 1 km.	6 (6.38)	6 (6.38)
1–5 km.	53 (56.38)	48 (51.06)
> 5 km.	35 (37.23)	39 (41.49)
Transportation to school		
Walk	1 (1.06)	1 (1.06)
Bicycle/Motorcycle	35 (37.23)	35 (37.23)
Personnel Vehicle	25 (26.60)	24 (25.53)
Car hire/School bus	31 (32.98)	31 (32.98)
Others	2 (2.13)	2 (2.13)
- Public bus		
- Public transportation		
Children with Disabilities	N= 15	N= 15
Level of Education		
Kindergarten	2 (13.33)	2(13.33)
Primary School	10 (66.67)	9(60.00)
High School	2 (13.33)	1(6.67)
Vocational Education	0 (0.00)	0(0.00)
Non-formal Education	1 (6.67)	3(20.00)
Others	0 (0.00)	0(0.00)
Distance from home to school		
< 1 km.	0 (0.00)	0(0.00)
1–5 km.	10 (66.67)	8(53.33)
> 5 km.	5 (33.33)	7(46.67)
Transportation to school		
Walk	0 (0.00)	0(0.00)
Bicycle/Motorcycle	5 (33.33)	5(33.33)
Personnel Vehicle	7 (46.67)	7(46.67)
Care hire/School bus	3 (20.00)	3(20.00)
Others	0 (0.00)	0(0.00)

### Children with chronic illness

For patients with chronic illnesses, 10.6% were in kindergarten, 68.1% were in primary school and 21.3% were in secondary school; none of them attended vocational education. After discharge, 10.6% adopted a modified study plan for non-formal education; 6.4% of parents lived less than 1 km away while 56.4% lived 1–5 km away from school and 41.5% were the same distance before and after attending the center. With regard to transportation, 37.2% went to school by bicycle or motorcycle, 25.5% by personal vehicle, and 33.0% by public transportation; the transportation data were almost the same before and after attending the center.

### Children with disabilities

For children with disabilities, while being treated and attending the center, 13.3% were in kindergarten, 66.7% in primary school, 13.3% in secondary school, and 6.7% attended an individualized education program (IEP); after discharge, 13.3% were in kindergarten, 6.7% were in primary school and 6.7% in secondary school, 73.3% attended an IEP. After discharge, none of the parents live closer to the center than 1 km, 53.3% lived 1–5 km away and 46.7% live more than 5 km away from the center. The distance was almost the same before and after being attending school at the center. For transportation, 33.3% go to school by bicycle or motorcycle, 46.7% by personal vehicle, and 20.0% by public transportation; the transportation percentages are almost the same before and after going to the center.

As shown in Table 3, the factors relating to modifications of the education plan of the school were subjected to logistic regression. Nearly 100% of the children as general patients, chronic patients, and patients with developmental problems continued their education. Patients with common illness did not adjust their education plan, although 10.6% of chronic patients and 73.3% of special patients did modify their plan. The number of chronic patients who modified their education plan was significantly greater than the number of general patients (OR = 0.05; 95% CI: 0.02-0.14). In terms of education level, 95.5% of primary school students and 53.2% of secondary school students had their education plan modified. The number of primary school students who modified their education plan was significantly higher than those at kindergarten level (OR = 2.08; 95% CI: 0.95-4.55).

**Table 3.** Factors related to modification of the education plan.

	Modification of education plan	Non-modification of education plan	Odds Ratio (95%CI)	*p* value
	N (%)	N (%)	N (%)	N (%)
**Children’s illness condition**				
Acute Illness (N = 90)	0 (0)	90 (100)	1	<.0001 *
Chronic Illness (N = 95)	10 (10.52)	85 (89.48)	0.05 (0.02 - 0.14)	
Disabilities (N = 15)	10 (66.66)	5 (44.44)	0.32 (0.06 - 1.75)	
**Education level**				
Kindergarten	0 (0)	36 (100)	1	0.0140 *
Primary School	5 (3.75)	127 (95.48)	2.08 (0.95 - 4.55)	
Secondary School	15 (46.80)	17 (53.20)	5.14 (2.06 - 12.83)	
**Length of hospital stay (days)**				
<5 days (N = 35)	0 (0)	30 (100)	1	0.0392 *
5–20 days (N = 111)	5 (2.28)	106 (97.72)	1.29 (0.81-2.06)	
>20 days (N = 69)	15 (21.73)	44 (63.76)	2.217 (1.172-4.194)	

**Notes:** Percentages are shown in a row. [1] The *p* value was based on logistic regression. [2] No 95%CI was considered. *significant difference ( *p* < 0.05)

As recorded in Table 4, parents’ opinions about each question across all types of illnesses are different. Considering the topic of learning, 74.7% of general patients, 69.2% of chronic patients, and 66.7% of disabled patients stated that the subjects at the center were interesting while 24.2% of general patients, 30.8% of chronic patients, and 33.3% of disabilities patients strongly agreed with this. More than half of the children enjoyed learning new things at the center (75.8% of patients with common illnesses, 70.2% of chronically ill patients and 73.3% of disabled patients). In terms of patients overall, 26.5% (23.1%, 29.8%, and 26.7% for those with common illness, those with chronic conditions and disabled children, respectively) strongly agreed that that learning at the center was enjoyable. In terms of looking forward to receiving services from the center, about 69.0% of all patients (73.6% of general patients, 65.9% of chronic patients, and 60.0% of patients with disabilities) agreed with this issue; and 22.2% of all patients (22.0% of general patients, 20.0% of chronic patients, and 22.0% of disabled patients) strongly agreed with this issue. Regarding the proper facilities supporting learning environment at the center, about 69.5% of all patients (71.4% of general patients, 67.0% of chronic patients, and 73.3% of special patients) agreed with this issue; and 31.9% of all patients (28.3% of general patients, 34.2% of chronic patients, and 35.1% of special patients) strongly agreed with this issue. In terms of advancement, varieties and adequacy of the study plan of the center, about 60.9% of all patients (65.6% of general patients, 57.7% of chronic patients, and 59.2% of special patients) agreed with this issue; and 30.0% of all patients (27.5% of general patients, 33.0% of chronic patients, and 26.7% of special patients) strongly agreed with this issue. The students also reported a variety of learning materials used in the classroom: 70.5% of patients used textbooks, (63.4% of general patients, 75.53% of chronic patients, and 80.0% of special patients) 76.0% of patients (78.0% of general patients, 80.8% of chronic patients, and 33.3% of special patients) used computer/notebooks or laptops, 48.0% of patients (52.8% of general patients, 47.9% of chronic patients, and 20.0% of special patients) used DVD/CVD and 17.0% of patients (14.3% of general patients, 19.2% of chronic patients, and 20.0% of special patients) used the Mobile Education Kit (MEK) eBooks, web-links, blogs/technical papers and mobile apps. MEK was made to develop a practical teaching and learning environment.

**Table 4.** Satisfaction with service at the Learning Center.

Degree of Satisfaction	Common Illness, n (%)	Chronic Illness, n (%)	Disabilities, n (%)	Total
Total	91 (45.5)	94 (47)	15 (7.5)	200 (100)
I am learning interesting subjects at the center				
Strongly disagree	0 (0.00)	0 (0.00)	0 (0.00)	0 (0.00)
Disagree	1 (1.10)	0 (0.00)	0 (0.00)	1 (0.50)
Not sure	0 (0.00)	0 (0.00)	0 (0.00)	0 (0.00)
Agree	68 (74.73)	65 (69.15)	10 (66.67)	143 (71.50)
Strongly Agree	22 (24.18)	29 (30.85)	5 (33.33)	56 (28.00)
I enjoy learning new things at the center				
Strongly Disagree	0 (0.00)	0 (0.00)	0 (0.00)	0 (0.00)
Disagree	0 (0.00)	0 (0.00)	0 (0.00)	0 (0.00)
Not sure	1 (1.10)	0 (0.00)	0 (0.00)	1 (0.50)
Agree	69 (75.82)	66 (70.21)	11 (73.33)	146 (73.00)
Strongly Agree	21 (23.08)	28 (29.79)	4 (26.67)	53 (26.50)
During hospitalization, I look forward to receiving services at learning center.				
Strongly Disagree	0 (0.00)	0 (0.00)	0 (0.00)	0 (0.00)
Disagree	0 (0.00)	0 (0.00)	0 (0.00)	0 (0.00)
Not sure	4 (4.40)	11 (11.70)	3 (20.00)	18 (9.00)
Agree	67 (73.63)	62 (65.96)	9 (60.00)	138 (69.00)
Strongly Agree	20 (21.98)	21 (22.34)	3 (20.00)	44 (22.00)
The atmosphere and facilities make me want to learn				
Strongly Disagree	0 (0.00)	0 (0.00)	0 (0.00)	0 (0.00)
Disagree	0 (0.00)	0 (0.00)	0 (0.00)	0 (0.00)
Not sure	1 (1.10)	0 (0.00)	0 (0.00)	1 (0.50)
Agree	65 (71.43)	63 (67.02)	11 (73.33)	139 (69.50)
Strongly Agree	25 (27.47)	31 (32.98)	4 (26.67)	60 (30.00)
The material is a variety of up-to-date learning material available for me				
Strongly Disagree	0 (0.00)	0 (0.00)	0 (0.00)	0 (0.00)
Disagree	0 (0.00)	0 (0.00)	0 (0.00)	0 (0.00)
Not sure	2 (2.20)	0 (0.00)	0 (0.00)	2 (1.00)
Agree	66 (72.53)	65 (69.15)	10 (66.67)	141 (70.50)
Strongly Agree	23 (25.27)	29 (30.85)	5 (33.33)	57 (28.50)
What types of learning materials are used at the center? (You may choose more than one answer)				
Fundamental textbook/Story book	58 (63.74)	71 (75.53)	12 (80.00)	141 (70.50)
Computer/notebook/laptop	71 (78.02)	76 (80.85)	5 (33.33)	152 (76.00)
DVD/VCD	48 (52.75)	45 (47.87)	3 (20.00)	96 (48.00)
Mobile Education Kit	13 (14.29)	18 (19.15)	3 (20.00)	34 (17.00)
Others	8 (8.79)	2 (2.13)	0 (0.00)	10 (5.00)
You are satisfied with the learning center				
Very dissatisfied	1 (1.10)	0 (0.00)	0 (0.00)	1 (0.50)
Dissatisfied	0 (0.00)	0 (0.00)	0 (0.00)	0 (0.00)
Moderately satisfied	4 (4.40)	4 (4.26)	1 (6.67)	9 (4.50)
Very satisfied	49 (53.85)	50 (53.19)	7 (46.67)	106 (53.00)
Extremely satisfied	37 (40.66)	40 (42.55)	7 (46.67)	84 (42.00)
Did you get further education after leaving the hospital?				
Yes	91 (100.00)	94 (100)	15 (100)	200 (100)
No	0 (0.00)	0 (0.00)	0 (0.00)	0 (0.00)
For those who continue their education, the center encouraged you to continue studying.				
Not at all	0 (0.00)	0 (0.00)	0 (0.00)	0 (0.00)
A little	0 (0.00)	1 (1.06)	1 (6.67)	2 (1.00)
Moderate	20 (21.98)	19 (20.21)	1 (6.67)	40 (20.00)
Very much	46 (50.55)	46 (48.94)	5 (33.33)	97 (48.50)
Extremely	25 (27.47)	27 (28.72)	8 (53.33)	60 (30.00)

Regarding satisfaction with services at the center, about 53.0% of all patients (66.5% of general patients, 55.6% of chronic patients, and 46.1% of special patients) were highly satisfied with the services; and 42.0% of all patients (40.7% of general patients, 42.6% of chronic patients, and 46.7% of special patients) were extremely satisfied. After completing education program at the center, about 100.0% (100.0% of general patients, 100% of chronic patients, and 100% of disabled patients) continue further education. About 20.0% (22.0% of general patients, 20.2% of chronic patients, and 6.7% of disabled patients) said that the center encouraged them to continue further education. About 48.5% (50.5% of general patients, 49.0% of chronic patients, and 33.3% of disabled patients) rated their satisfaction towards the learning center at a high level; and about 30.0% (27.5% of general patients, 28.7% of chronic patients, and 53.3% of special patients) rated at very high level.

## Discussion

From the study of the operation of the learning center in the hospital at QSNICH, most child patients have chronic diseases because QSNICH is the only public children’s hospital that serves child patients at the tertiary level and provides care to pediatric patients referred from across Thailand. The most common chronic diseases found in pediatric patients at QSNICH are cancer, heart disease and neurological disease. The results of this study are similar to those of previous studies ^10, 15, 16^ at tertiary hospitals. Common illnesses in children are gastrointestinal disease, infections, and respiratory illness. Patients with disabilities were children with ADHD, intellectual deficits, learning disabilities and autism. Compared to a previous study ^10^, the number of disabled children at the learning center increased because of the increased medical emphasis on holistic service both physically and developmentally. Teachers at the learning center can follow published guidelines ^17, 18^ and utilize a project evaluation manual developed in 2016. The average age of patients with chronic illnesses was 11.22 years, while common illnesses occurred mostly in children with an average age of 9.7 years. followed by an average age of children with disabilities at 9.69 years. After discharge, children in kindergarten were ready to continue their education in primary school.

### Key findings

Education management should be supported and promoted to encourage students to continue their studies in the regular education system. If a student’s treatment schedule causes them to miss classes, the learning center will modify their study plan by providing extra classes during hospitalization, allowing them to interact with teachers at the learning center. Teachers at the learning center coordinate their curriculum with that of regular school teachers. After being discharged, the children continue their education in the same class. Education ^19, 20^, as well as social and behavioral interactions, are very important for children during hospitalization.

### Limitations

The clinical demographic groups in this study were only selected from children at the Queen Sirikit National Institute of Child Health. The respondents from other institutes should be included as well to increase diversity through a variety of contexts. Future studies should involve learning centers in different types of hospitals such as primary, secondary and tertiary care facilities in each region in Thailand since they all provide different levels of services for patients with different needs.

### Implications

Learning via social media ^20– 22^ are some of the available channels allowing the children to keep in touch with friends and teachers, reduce stress and the feeling of being left out, and prevent social problems after returning to school. They can also share experience at the hospital, turn crisis situations into opportunities and add their own perspectives and ideas about making life better for hospitalized children. Comprehensive ^19, 22^ planning will enhance children’s skill in social interaction, education and life adjustment.

Our study found that ten chronically ill children were evaluated, and their non-formal education plans were adjusted appropriately. Similarly, ten disabled children enrolled in IEPs, which provide customized curricula for special needs children that are approved by family and school. Chronically ill and disabled children seem to benefit most from the statistically significant modifications in their education plans. Children with illnesses at the secondary level will be enrolled in non-formal education programs. The study plan of children who stay in the hospital for more than 20 days is more likely to be adjusted than for a five-day period of hospitalization. Other factors studied include the demographics, economic status, occupation and distance from home to school; however, these showed no statistical significance in curriculum adjustment.

From the results of our study, it is clear that we should consider evaluating the appropriateness of an education plan by having the teachers at the hospital learning center work closely with regular school teachers to perform long-term monitoring and appropriate planning. The study of parents’ attitudes towards their children’s illnesses and education found that most think chronic diseases are very severe, but parents of special needs children and those with common illnesses do not think this is a serious issue and give more attention to education of their children. Parents of chronically ill children and those with common ailments always inform the school when the patients will be absent, but only about half of the parents of children with disabilities do so because they already have individual education plans and they know that school teachers and administrators will arrange appropriate plans to accommodate the students. Social adjustment ^19– 21, 23^ is a major concern of parents, most of whom have little confidence that schools can provide adequate services in this area to sick children. On the other hand, parents of disabled children feel moderately to highly secure that the teaching staff at special needs schools will involve their children in building good relationships with friends. In order to arrange learning activities for sick children, teachers must have broad knowledge including educational psychology and a good understanding of the medical conditions because each child has a different set of physical and mental states that require different educational approaches. Most children worry about their illness and this can strongly affect their lifestyle ^18, 21, 24^ so appropriate guidance is also needed in addition regular coursework.

### Recommendations

Mobile learning center are good for child patients because their environments are flexible, and they are less likely to feel trapped in a hospital room. Children who must stay in bed will be offered bedside teaching programs. The mobility of educational settings has been greatly improved through use of electronic devices to provide the same standards as obtained in more formal schools. After analyzing the satisfaction result at the learning center, the highest score was from 4.21 to 5.00 (mean = 4.28, SD = 0.62). Considering each item, the respondents reported that having up to date teaching methods and innovative learning activities ranked as the most satisfying features while interesting subject matter and knowledge were second highest because of using technology to help with learning like Electronic Distance Learning Television computer programs to meet international standards as stated by the Ministry of Education.

The third highest score was enjoyment in the educational entertainment activities, while the fourth ranking went to setting up an environment most conducive to learning. (mean = 4.18, SD = 0.79). Having enough modern learning materials came in fifth. Compared to previous studies ^10, 15^, our results demonstrated the importance of adjustments in educational programs as needs changed in line with new teacher guidelines, evaluations of the work of the center, greater involvement of learning materials and technology as standard requirements of the course, and ways to reduce problems between teachers and students with regard to using mobile education. However, new materials, equipment and programs still need to be developed. Based on studies in other countries ^22, 24, 25^, we concluded that the best educational environment can be achieved with a mix of technology and conventional teaching. The unique, multidisciplinary educational system for sick children in a hospital needs to be supported by hospital, family, and community in concert with local laws. Everyone can support continuing education as a holistic system, reducing the burden on families and society and meeting the purpose of building valuable infrastructure to develop the country’s human resources into the future.

## Conclusions

All the child patients at the learning center, QSNICH, are able to continue their education. On average, 20% of child patients need to modify their education plan according to their condition. This is most evident among children with chronic illness and disabilities, those at the primary and secondary school levels, and children requiring prolonged hospitalization. The majority of parents have appropriate attitudes about the treatment and education of their children and the children gave the programs their highest rating showing extremely high satisfaction with the service at the learning center in the hospital.

## Data availability

### Underlying data

Figshare: Data record of the survey. https://doi.org/10.6084/m9.figshare.8479352.v2 ^14^.

This project contains the raw survey responses of each participant.

### Extended data

Figshare: English Survey.pdf. https://doi.org/10.6084/m9.figshare.8208338.v2 ^13^.

This project contains the following extended data:

• English Survey.pdf
• QSNICH Princess IT_Version 1.0_09May2017.pdf (survey in Thai)

Data are available under the terms of the Creative Commons Attribution 4.0 International license (CC-BY 4.0).
