# Supplementary material for: Effectiveness of the Hospital Learning Center (Queen Sirikit National Institute of Child Health): Satisfaction with service and parents’ attitudes towards children’s illness
Source: F1000Res. 2019 Sep 10;8:1616. [Version 1] doi: 10.12688/f1000research.18846.1 (PMC7218477; doi:10.12688/f1000research.18846.1)
Supplement: Peer review history

# Peer review: Reviewer response for version 1

**DOI:** 10.5256/f1000research.20653.r62258

**Published:** 2020-05-12

**Authors:** Banchaun Benjasuwantep

**Affiliations:** 1 Department of Pediatrics, Faculty of Medicine, Srinakharinwirot University, Nakhon Nayok, Thailand

The study is about hospital learning center that is interesting and important for children to continue their learning outside school. However, I have some suggestions: There are 200 data in the research rather than 400 people. The author can explain that 200 children included in the research and 200 parents were interviewed.  

**Introduction: **

- The author should review other countries' hospital learning centers and their effectiveness in education.

 

**Method: **

- The method is short. The author should give more information about the questionnaire especially the relationship between parents and children and children's personal information. The author should explain how it relates to education (the objective of the research) 
- It will make readers clearly understand and follow the article. It is unclear about the hospital learning center such as the reasons teachers modify children's education plans and if they contact children's school teachers?

**Result: **

- The author should put subtitle before changing the subject, for example, puts words like "education" before according to table 1. The readers will clearly follow the article.

Is the work clearly and accurately presented and does it cite the current literature?

Partly

If applicable, is the statistical analysis and its interpretation appropriate?

I cannot comment. A qualified statistician is required.

Are all the source data underlying the results available to ensure full reproducibility?

Partly

Is the study design appropriate and is the work technically sound?

Yes

Are the conclusions drawn adequately supported by the results?

Yes

Are sufficient details of methods and analysis provided to allow replication by others?

Partly

Reviewer Expertise:

Developmental behavioral pediatrician

I confirm that I have read this submission and believe that I have an appropriate level of expertise to confirm that it is of an acceptable scientific standard.

# Peer review: Reviewer response for version 1

**DOI:** 10.5256/f1000research.20653.r61226

**Published:** 2020-03-26

**Authors:** Savithiri Ratnapalan

**Affiliations:** 1 Department of Paediatrics, University of Toronto, Toronto, Canada

**General comments**

 The authors sampled children and their parents who had attended a hospital learning center to identify practice patterns, satisfaction and impact. A very good study highlighting the importance of educating hospitalized children and the impact on children and families. The paper needs to be edited again for grammar and spelling.

**Specific Comments**

 Table 1 - How often do you explain the lesson to your child? Almost never repeated twice. I believe it should read -Almost always.

 The discussion part has to be rearranged. After results, the authors can discuss their finding, then either use implications, limitations and conclusions.

 The first part of the discussion can be moved to the methodology section so that the readers know what common diseases and chronic diseases are being discussed.

 Stat discussing the findings first - Your section on implications and recommendations does that but needs to be edited to discuss the study finding.

 The key messages can be given in a separate text box as bullet points:

  

- Education of hospitalized children should be supported and promoted to encourage students to continue their studies in the regular education system.

 

- Teachers at hospital learning centers should coordinate their curriculum with regular school teachers and modify the curriculum to suit the child’s educational need.

 

- Mobile learning centers can be used for some children to teach at the bed side.

 

- Electronic devices can be used to enhance the education of hospitalized children

Is the work clearly and accurately presented and does it cite the current literature?

Yes

If applicable, is the statistical analysis and its interpretation appropriate?

I cannot comment. A qualified statistician is required.

Are all the source data underlying the results available to ensure full reproducibility?

Yes

Is the study design appropriate and is the work technically sound?

Yes

Are the conclusions drawn adequately supported by the results?

Yes

Are sufficient details of methods and analysis provided to allow replication by others?

Yes

Reviewer Expertise:

Education of hospitalized children, Medical Education,  Paediatric Emergency care

I confirm that I have read this submission and believe that I have an appropriate level of expertise to confirm that it is of an acceptable scientific standard, however I have significant reservations, as outlined above.

# Peer review: Reviewer response for version 1

**DOI:** 10.5256/f1000research.20653.r57247

**Published:** 2019-12-10

**Authors:** Issarapa Chunsuwan

**Affiliations:** 1 Department of Pediatrics, Faculty of Medicine, Developmental and Behavioral Pediatrics, Thammasat University (TU), Pathum Thani, Thailand

The effort to help children with learning center while at the hospital is impressive and the result revealed that many children and families were satisfied with the hospital learning center.

 I have some suggestions:

- The title should be adjusted. The word effectiveness should be used to explain what effect the child has when they attended the learning center, such as getting a better score, better adaptation when go back to school. In this research, there is no mention of that issue, but mainly as a study of satisfaction.
- Authors should explain more about the teaching and learning at the center, such as setting and management, teachers: qualification, frequency of attendance, curriculum. Such things would help readers understanding more about the center and could be reproducible.
- Table 2: It would be better if authors show or explain more about the concept of what you want to convey? Displaying the percent before and after of each items do not show clear picture. Author may use statistics to analyze or discuss more about Table 2 in the discussion part
- Table 3: please check the odd ratio in the illness condition again if the first row (acute illness) is a reference value (odd is equal to 1). The odd value of chronic illness and disability should be greater than 1.
- Table 4: The authors try to show the comparison results between 3 groups in degrees of satisfaction. The table should show statistical analysis to compare whether there are differences or not, for clearer picture.
- In discussion part, the author should explain further as specified in the objectives of the research. For example, discuss about satisfaction for learning center, factors effect modification of educational plan.  

Is the work clearly and accurately presented and does it cite the current literature?

Partly

If applicable, is the statistical analysis and its interpretation appropriate?

Partly

Are all the source data underlying the results available to ensure full reproducibility?

Yes

Is the study design appropriate and is the work technically sound?

Partly

Are the conclusions drawn adequately supported by the results?

Partly

Are sufficient details of methods and analysis provided to allow replication by others?

Partly

Reviewer Expertise:

Developmental and behavioral pediatrics

I confirm that I have read this submission and believe that I have an appropriate level of expertise to confirm that it is of an acceptable scientific standard.
